# Retirement, Cognitive Health, and Neighborhood Environment: Evidence from the Health and Retirement Study

**DOI:** 10.1093/geroni/igaf122.1584

**Published:** 2025-12-31

**Authors:** Debasree Das Gupta, Uma Kelekar, David Wong, Kallol Kumar Bhattacharyya, Jeein Law (Jang

**Affiliations:** Utah State University, Logan, Utah, United States; Marymount University, Arlington, Virginia, United States; George Mason University, Fairfax, Virginia, United States; The University of Memphis, Memphis, Tennessee, United States; University of Massachusetts Boston, Boston, Massachusetts, United States

## Abstract

The mental retirement hypothesis suggests that retirement may lead to cognitive decline due to reduced intellectual engagement and decreased cognitive reserve. Engaging retirees in mentally stimulating environments is encouraged, particularly within their neighborhoods. However, current empirical research has not adequately examined how neighborhood factors influence the relationship between retirement and cognitive health. To address this gap, we utilized data from two waves (2016-2018) of the Health and Retirement Study and analyzed whether perceived neighborhood environment moderates the association between retirement and cognitive health over a two-year follow-up period, after adjusting for covariates (retirement duration, baseline sociodemographic, socioeconomic, and health factors). The study sample (n = 2,040) consisted of non-institutionalized adults, age 51-70 years with no prior history of stroke or baseline cognitive impairment. Perceived neighborhood environment was measured using self-reported data on four separate neighborhood indicators that included social cohesion (e.g. friendliness/trustworthiness of neighbors), physical disorder (e.g. graffiti/vacant houses in neighborhood), social ties (friends/relatives in neighborhood), and safety. In the adjusted regression models, mean cognitive function at follow-up was significantly lower (β=-6.79; 95%CI=-13.06,-0.52) among those retired, compared to those fully or partly engaged in the workforce. However, this gap decreased by 1.02 units (β=-1.02; 95%CI=-0.06,-1.99) with each unit increase in perceived social cohesion. The remaining three neighborhood indicators did not significantly moderate the retirement—cognitive health relationship. Our findings indicate that greater social cohesion positively moderated the adverse relationship between retirement and cognitive health. Neighborhood resources that promote social and mental engagement may help protect the cognitive health of retirees.

